# Effect of Aging on Physicochemical Properties and Size Distribution of PET Microplastic: Influence on Adsorption of Diclofenac and Toxicity Assessment

**DOI:** 10.3390/toxics11070615

**Published:** 2023-07-14

**Authors:** Josipa Papac Zjačić, Stefani Tonković, Anamarija Pulitika, Zvonimir Katančić, Marin Kovačić, Hrvoje Kušić, Zlata Hrnjak Murgić, Ana Lončarić Božić

**Affiliations:** 1 Faculty of Chemical Engineering and Technology, University of Zagreb, Trg Marka Marulića 19, 10000 Zagreb, Croatia; jpapac@fkit.unizg.hr (J.P.Z.); stonkovic@fkit.unizg.hr (S.T.); pulitika@fkit.unizg.hr (A.P.); katancic@fkit.unizg.hr (Z.K.); mkovacic@fkit.unizg.hr (M.K.); zhrnjak@fkit.unizg.hr (Z.H.M.); abozic@fkit.unizg.hr (A.L.B.); 2 Department for Packaging, Recycling and Environmental Protection, University North, Trg dr. Žarka Dolinara 1, 48000 Koprivnica, Croatia

**Keywords:** microplastics, aging, polyethylene terephthalate, diclofenac, adsorption, toxicity

## Abstract

Microplastics (MPs) are detected in the water, sediments, as well as biota, mainly as a consequence of the degradation of plastic products/waste under environmental conditions. Due to their potentially harmful effects on ecosystems and organisms, MPs are regarded as emerging pollutants. The highly problematic aspect of MPs is their interaction with organic and inorganic pollutants; MPs can act as vectors for their further transport in the environment. The objective of this study was to investigate the effects of ageing on the changes in physicochemical properties and size distribution of polyethylene terephthalate (PET), as well as to investigate the adsorption capacity of pristine and aged PET MPs, using pharmaceutical diclofenac (DCF) as a model organic pollutant. An ecotoxicity assessment of such samples was performed. Characterization of the PET samples (bottles and films) was carried out to detect the thermooxidative aging effects. The influence of the temperature and MP dosage on the extent of adsorption of DCF was elucidated by employing an empirical modeling approach using the response surface methodology (RSM). Aquatic toxicity was investigated by examining the green microalgae *Pseudokirchneriella subcapitata*. It was found that the thermooxidative ageing process resulted in mild surface changes in PET MPs, which were reflected in changes in hydrophobicity, the amount of amorphous phase, and the particle size distribution. The fractions of the particle size distribution in the range 100–500 μm for aged PET are higher due to the increase in amorphous phase. The proposed mechanisms of interactions between DCF and PET MPs are hydrophobic and π–π interactions as well as hydrogen bonding. RSM revealed that the adsorption favors low temperatures and low dosages of MP. The combination of MPs and DCF exhibited higher toxicity than the individual components.

## 1. Introduction

Plastics are cost-effective, versatile materials that benefit society and improve people’s quality of life in many ways. The global plastic production reached 367 million tonnes in 2020, an increase of >80% over the last 20 years [1,2]. The most commonly used polymer materials for many different applications are polyethylene (PE), polypropylene (PP), polyvinyl chloride (PVC), polystyrene (PS), and polyethylene terephthalate (PET). PET is a low-cost, lightweight, and durable thermoplastic polyester that is mainly used to produce synthetic fibers for clothing as well as blow-molded bottles for water, soft drinks, juices and detergents, and other containers. The high consumption of plastic products and improper waste management practices result in large amounts of plastic waste entering the environment. In addition, single- or short-term-use plastic products exacerbate the issue of plastic pollution. Although PET is the most recycled plastic in the world, virgin PET amounts to more than 4 million tonnes in the European market [1]. In recent years, the issue of microplastics (MPs) has been brought into focus. MPs are plastic particles smaller than 5 mm, the occurrence of which in the aquatic environment is increasingly reported [3,4,5,6]. In addition, they can be divided into primary and secondary MPs. Primary MPs are produced in small dimensions and used in the formulation of various products such as cosmetics or cleaning agents, while secondary MPs originate from the deterioration of larger objects and plastic waste. Deterioration occurs during use or after the disposal of the plastic product. Many different stressors occur in the environment, such as sunlight, heat, cold, freezing, humidity, and water, which play a crucial role in the ageing and degradation of polymer materials [7,8] and fragmentation into MPs. The ageing of polymers causes damage that can be divided into (i) physical—reduction in molecular weight, impact strength, yield strength, elongation, and changes in gloss and color; and (ii) chemical—changes in the chemical structure and formation of surface functional groups, radicals, and small volatile compounds. Deterioration can be visible, in the form of cracks or crazing, and invisible, inside the material, becoming visible as actual fractures when stressed [9,10]. Cracks on the surface make the interior of the plastic accessible to further degradation, which eventually leads to embrittlement and fragmentation into small and micro-sized particles. Studies on the formation of secondary MPs are mainly concerned with photochemical degradation [11,12], while less attention is given to thermooxidative degradation [13]. The difference between these two processes is the formation of polymer radicals, which is initiated by UV irradiation and heat, respectively. Moreover, photooxidative degradation occurs only on the surface and subsurface layers of polymer, in contrast to thermooxidative degradation, which extends throughout the polymer material [14]. The thermooxidative degradation of PET proceeds by a chain-scission at ester linkages and the formation of carboxyl and vinylester end groups (Figure S1), which can play an important role in the sorption mechanism of various pollutants [15]. Transesterification of vinyl ester produces vinyl alcohol, which converts to volatile acetaldehyde as the main degradation byproduct (Figure S3). Other volatile degradation products such as carbon monoxide and dioxide, ethylene, acetylene, and water are formed in very small amounts at low temperatures (Figure S2) [16,17,18,19,20]. Due to their small size, large specific surface area, and high hydrophobicity, as well as the presence of proton donor and proton acceptor functional groups, MPs can adsorb pollutants and thus serve as transport vectors in the environment. Ageing leads to changes in physicochemical properties, i.e., changes in size and physical morphology as well as functional groups on the (micro)plastic surfaces, and is thus a key factor influencing the adsorption properties of MPs in the environment [21,22].

In this work, the effect of ageing on the physicochemical properties and size distribution of PET MPs, affecting the adsorption of diclofenac and the resulting toxicity to the freshwater algae *Pseudokirchneriella subcapitata* (*Selenastrum capricornutum*), was studied. To this end, PET was subjected to accelerated thermo-oxidative ageing at elevated humidity and with a controlled temperature regime in the laboratory [23,24,25]. The aged and untreated (pristine) materials were characterized using various techniques to investigate hydrophilicity, roughness, crystallinity, and oxygenated functional groups and to correlate the observed changes with the adsorption of diclofenac, a non-steroidal anti-inflammatory drug. Diclofenac was chosen because of its abundance in natural waters due to its widespread consumption and limited efficacy of elimination by conventional wastewater treatment, causing the presence of diclofenac even in drinking water sources [26]. It should be noted that diclofenac has been classified as a contaminant of emerging concern and was included in the previous Watch List of the EU Water Framework Directive [27], while recent indications suggest that diclofenac will be included in the list of priority substances in the next prioritization. The selection of the freshwater algae *Pseudokirchneriella subcapitata* for toxicity testing is based on the fact that microalgae are the main producers of organic matter in water bodies and are the primary producer at the trophic level as well. Their importance as an indicator of pollution is widely known due to their high sensitivity, simple cultivation process, and short growth period [28]. Hence, the freshwater algae *Pseudokirchneriella subcapitata* was used in many studies for testing the toxicity of a vast array of environmental pollutants, particularly due to its high sensitivity [29,30].

## 2. Materials and Methods

### 2.1. Materials

Pharmaceutical diclofenac (DCF) in the form of sodium salt (C_14_H_11_Cl_2_NNaO_2_, p.a.) was purchased from Sigma-Aldrich, Burlington, MA, USA. The auxiliary chemicals sulfuric acid (H_2_SO_4_, 96%) and sodium hydroxide (NaOH, p.a.) used for the adjustment of the initial pH value were purchased from Kemika d.o.o., Zagreb, Croatia. Calcium chloride (CaCl_2_, 96%), purchased from Kemika d.o.o., Zagreb, Croatia, was used to ensure a certain ionic strength of the MP suspension in the DCF solution. PET ((C_10_H_8_O_4_)_n_) used for the preparation of MP samples was sourced from a commercial clear PET bottle (artesian water Jana, Zagreb, Croatia); thickness 250 μm, and PET foils (Uredski sistemi d.o.o., Zagreb, Croatia), thickness 700 μm. Methanol (CH_3_OH, HPLC grade, Fluka, Buchs, Switzerland), formic acid (HCOOH, HPLC grade, JT Baker, Switzerland), and ultra-pure water (prepared with a MiliPore Sigma water purification system, Merck Millipore, Burlington, MA, USA) were used as components of the mobile phase for chromatographic analysis, as well as for the preparation of DCF stock solution. All chemicals and nutrients for toxicity bioassays were purchased from Microbiotest, Ghent, Belgium.

### 2.2. Procedures

The PET samples prepared from bottles and foils are referred to as PET_B and PET_F, respectively, and were submitted to the accelerated thermo-oxidative ageing at elevated humidity and controlled temperature using a UE laboratory oven (Memmert, Schwabach, Germany) and a refrigerated cabinet (Končar—Kućanski Aparati d.d., Zagreb, Croatia). The samples were aged for 14, 28, and 42 days. The daily weathering cycle consisted of three phases of thermooxidative treatment: (1) 16 h at 70 °C in dry air; (2) 4 h at 70 °C in humid air; and (3) 2 h at −18 °C. The samples were then designated according to the days of ageing as PET_B_0 and PET_F_0 (pristine) and PET_B_14, PET_B_28, PET_B_42, and PET_F_14, PET_F_28, PET_F_42 (aged). The pristine and aged PET samples were then ground into MPs using a cryogenic ball mill (Retsch, Haan, Germany). The mill charge was cooled with liquid nitrogen for the duration of the grinding process. To investigate the effects of ageing on fragility and brittleness, grinding was performed with stainless steel balls in three cycles as follows: precooling (*f* = 5 s^−1^; *t* = 1 min) and grinding (*f* = 25 s^−1^; *t* = 1 min), with the intercooling between each grinding cycle (*f* = 5 s^−1^; *t* = 30 s). The obtained MPs were then sieved with five sieves of different mesh sizes, using the Sieve Shaker AS 200 control (Retsch, Germany) to separate and analyze the particle size and mass distribution. The following fractions were obtained: >500 µm, 400–500 µm, 300–400 µm, 200–300 µm, 100–200 µm, and <100 µm, and their mass fractions were determined gravimetrically.

Adsorption and toxicity experiments were performed with both pristine (PET_B_0, PET_F_0) and aged MPs (PET_B_42, PET_F_42). The adsorption experiments were performed with an OLS Aqua Pro orbital shaker (Grant, Royston, UK). The DCF solution (0.05 mM) was prepared with ultra-pure water (*ρ* = 18 MΩ × cm, EMD Millipore, Burlington, MA, USA). MP with different loading (250 mg L^−1^, 500 mg L^−1^, and 750 mg L^−1^) and calcium chloride (0.01 M) were added to the DCF solution. The adsorption experiments were conducted for 24 h according to the Full Factorial Design (FFD) experimental plan (Table 1), in combination with response surface modelling (RSM) to study the adsorption of DCF on MPs as a function of the two studied independent parameters (temperature and MP loading). The obtained results were processed and analyzed using the software packages Design Expert 10.1 (StatEase, Minneapolis, MN, USA) and STATISTICA v.14 (TIBCO, Palo Alto, CA, USA). The goodness-of-fit of the RSM models was estimated on the basis of the coefficient of determination (*R*^2^) and analysis of variance (ANOVA). The samples of the adsorption tests were filtered through a 0.45 µm PTFE filter prior to submission to the HPLC analysis.

### 2.3. Analysis and Characterization

Prior to the grinding and obtaining of MPs of specific size fractions, the PET foils were characterized using various instrumental techniques. ATR-FTIR spectroscopy (Spectrum One, Perkin Elmer, USA) was used to determine the carbonyl index of pristine and aged PET samples. The surface wettability was assessed by water contact angle measurements using the OCA20 goniometer (Data Physics, Filderstadt, Germany). Thermal characterization of pristine and aged samples was performed by differential scanning calorimetry (DSC) using a DSC 823eT calorimeter (Mettler Toledo, Greifensee, Switzerland) and thermogravimetric analysis (TGA) was carried out using a Q500 TGA (TA Instruments, New Castle, DE, USA). DSC was used to determine the enthalpy of crystallization, while TGA analysis was performed to determine the water uptake. The microstructural morphologies of the pristine and aged samples were analyzed by using a Vega III scanning electron microscope (SEM) (Tescan, Brno, Czech Republic). The zeta potential of the MPs was measured using a Zetasizer Ultra (Malvern Panalytical, Worcesterhire, UK). Details on the principles of the performed characterization techniques are provided in Text S1 (Supplementary Materials).

To investigate the adsorption of DCF on PET MPs, samples were taken after 24 h and filtered through a PTFE filter with a 0.45 µm pore size (Chromafil, Macherey-Nagel, Düren, Germany) prior to the HPLC analysis. The DCF concentration was determined using high-performance liquid chromatography coupled with a UV/DAD detector (Shimadzu LC-20 series, Kyoto, Japan) at the UV wavelength 276 nm. A 250 mm × 4.6 mm, 5 µm Nucleosil C18 column (Macherey-Nagel, Germany) was used to separate DCF. For this purpose, mobile phase consisting of 0.1% formic acid and methanol in a 30:70 ratio at a flow rate of 1.0 mL/min in an isocratic mode was used, as detailed in our previous study [31]. The sample injection volume was 50 µL. All samples were analyzed in triplicate and average values were reported (reproducibility was >98.3%). The extent of adsorption was monitored by determining the difference in DCF concentration in the initial solution and in the samples after 24 h.

### 2.4. Toxicity Bioassay

The aquatic toxicity assays and corresponding calculations were performed according to the standard freshwater algal growth inhibition test with the unicellular green algae *Pseudokirchneriella subcapitata* (*Selenastrum capricornutum*), using the procedure disclosed in ISO 8692:2012. For this purpose, an Algaltoxkit F (Microbiotest, Gent, Belgium) was used. Firstly, the toxicity of individual constituents, i.e., CaCl_2_, DCF, and pristine and aged PET MPs with a particle size distribution of 100–500 µm, was determined. Secondly, the effect of the combined toxicity of DCF and MPs was investigated to elucidate the combined effects. The optical density was first corrected for the values attributable to the MP particles in order to distinguish them from the optical density attributable to the algal cells. The test batches were incubated for 72 h, and the cell density in each test solution was measured every 24 h. For that purpose, a Jenway 6200 spectrophotometer (Fisher Scientific, Waltham, MA, USA) with a holder for 10 cm optical path length cells was used. More detailed insight into the calculations performed is provided in Text S3 (Supplementary Materials).

## 3. Results

### 3.1. FTIR Analysis

The FTIR spectra (Figure S4, Supplementary Materials) show characteristic bonds of the investigated PET bottles and foils before and after thermooxidative aging, identifying the chemical composition and conformations of functional groups. Carboxyl groups were observed at 1711 cm^−1^, vibrations of C=O at 1233 cm^−1^ with C-O stretching bonds at 718 cm^−1^, and vibrations of C-C bending/stretching bonds of the aromatic ring at 1404 cm^−1^ as well as C-H vibrations of -CH_2_ groups of ethylene glycol in *gauche* or *trans* conformation at 1376 cm^−1^ and 1343 cm^−1^, respectively. Moreover, further important vibrations observed are the symmetric (1090 cm^−1^) and asymmetric (927 cm^−1^) stretching of the oxyethylene bond (O-C) from the O-CH_2_ group of ethylene glycol in *trans* and *trans* conformation [32,33]. These bonds are important to identify the amorphous and crystalline phases in the polymer PET since the *trans* conformation is found exclusively in the crystalline phase and the *gauche* conformation in the amorphous phase [34,35]. It is important to observe these phase changes during thermooxidative ageing as they are the result of structural transformation within the polymer molecules.

Analysis of the FTIR spectrum shows that the absorbance intensity of some bonds in the polymer remains unchanged during thermooxidative ageing, e.g., the absorbance of the aromatic ring at 1404 cm^−1^ [20]. Therefore, the changes in the absorbance of the characteristic groups with respect to the absorbance of the aromatic ring were calculated, and their ratios are given in Table 2. The absorbance ratio for the C=O group originating from the carboxyl groups increases with ageing for both types of PET samples. The absorbance increase in the C=O bonds is 3.9% for PET bottles after 42 days of aging and 20.6% for PET foils. Thus, the absorbance changes indicate changes in the structure of the PET molecules, i.e., they confirm that thermooxidative degradation of the PET molecules has occurred. In addition, vinyl ester, vinyl alcohol, acetaldehyde, and other volatiles are presumably formed in the initial stage of thermooxidative decomposition, which is a random process that leads to the cleavage of the PET molecule (Scheme S3). In fact, it has been confirmed in the literature that the absorbance intensity of the carbonyl groups decreases in the initial phase of decomposition and increases again after a much longer aging phase [20,35,36,37]. The calculated C.I. values for both bottles and foils follow the increasing trend until 28 days of aging and then start to decrease for both samples.

PET degradation is accompanied by conformational changes; a slight decrease in absorbance intensity for the *trans* conformation (9%) and an increase for the *gauche* conformation (16%) can be observed for PET bottle samples as a result of ageing, while for PET foils, the ratio remains almost unchanged (Table 2). It is assumed that this effect is due to the difference in thickness of the samples tested, as the foils are three times thicker and changes in the bulk of the polymer are difficult to observe by FTIR. Furthermore, when considering the *gauche* and *trans* positions of the oxyethylene group (O-C) from the O-CH_2_ group of ethylene glycol at 972 and 1090 cm^−1^, it is seen that the *gauche* and *trans* conformation increases until 28 days of aging and then decreases for both samples, although at the end, the *trans* conformation decreases by 9% and 5%, respectively. As indicated, the decrease in *trans* and increase in *gauche* conformation with ageing points to a structural transformation that is less ordered as a result of the cleavage of the PET polymer molecules. Namely, as the degree of degradation increases, the crystalline phase decreases because the segments of smaller molecules are not large enough to form a crystalline phase, although the crystallization process alone is a spontaneous process and is energetically more favorable [38].

### 3.2. DSC Analysis

The main purpose of characterizing the PET foils by DSC was to estimate the degree of crystallinity during the aging process. From the DSC results, the melting temperatures (*T*_m_) as well as the glass transition temperatures (*T*_g_) remained the same with aging (for both samples) (Table 3), but significant changes in the crystallization temperature (*T*_c_) were observed for PET bottle samples. In addition, the enthalpy of melting (Δ*H*_m_) and enthalpy of crystallization (Δ*H*_c_) changed significantly with aging compared to control, pristine PET samples (Table 3). One of the most important properties of semi-crystalline polymers such as PET is that they are two-phase systems, as they contain both crystalline and amorphous phases [39]. Therefore, the degree of crystallinity (*X*) for the studied samples was determined according to the following equation:

(1) $$X=\frac{\Delta H_{m}-\Delta H_{\mathrm{cc}}}{\Delta H_{m100}}\times 100\%$$

where Δ*H*_m_ is the enthalpy of melting (Jg^−1^), Δ*H*_cc_ is the enthalpy of cold crystallization (Jg^−1^), and Δ*H*_m100_ is the melting enthalpy for 100% crystalline PET (140 Jg^−1^ [40,41]).

From the results obtained, the degree of crystallinity decreases in both types of samples compared to the pristine samples. The decrease in crystallinity for PET foils is 17%, and for PET bottles, it is 9%. The decrease in the degree of crystallinity is due to the cleavage of macromolecules during thermooxidative aging. The degradation process produces shorter (smaller) molecules, and a larger number of shorter molecules have more ends per unit volume than long molecules. They have greater mobility than the segments of macromolecules. Therefore, a larger free volume indicates a higher fraction of the amorphous phase [35]. The results indicate that for both types of PET samples, the decomposition of polymer macromolecules occurs during thermooxidative aging when the degree of crystallinity decreases.

### 3.3. Surface Analysis

Contact angle measurement was used to characterize the surface of PET bottles and foils to assess changes during thermooxidative aging. This is determined by the changes in the intermolecular interaction between the surface of the samples and the water; the results are shown in Table 4. The value of the contact angle (*θ*) increased from 69° (PET_B_0) to 76° (PET_B_42) for PET bottles and from 61° (PET_F_0) to 72° (PET_F_42) for PET foils, corresponding to increases of 10% and 18%, respectively. From these results, it can be concluded that the contact angle increases with thermooxidative aging for both PET samples. The increase in contact angle indicates an increase in surface polarity due to a change in the structural properties of the PET molecules, i.e., the formation of functional end groups, oxy groups on polymer molecules, and radicals. Therefore, a higher contact angle means an increase in the hydrophobicity of the surface of PET polymer. Materials that have a contact angle of less than 90° have good wetting properties and are considered hydrophilic materials, while materials with an angle greater than 90° have very weak wetting properties and are considered hydrophobic materials [42]. In this work, the process of adsorption of substances (i.e., DCF), which have potentially harmful effects on the aquatic environment, on the surface of PET MPs after aging is studied. Therefore, it was important to identify the changes in the structure of PET that contribute to the change in surface properties and to determine whether they contribute to the increase in the adsorption of toxic substances from water on the surface.

### 3.4. SEM Analysis

**Figure 1:**
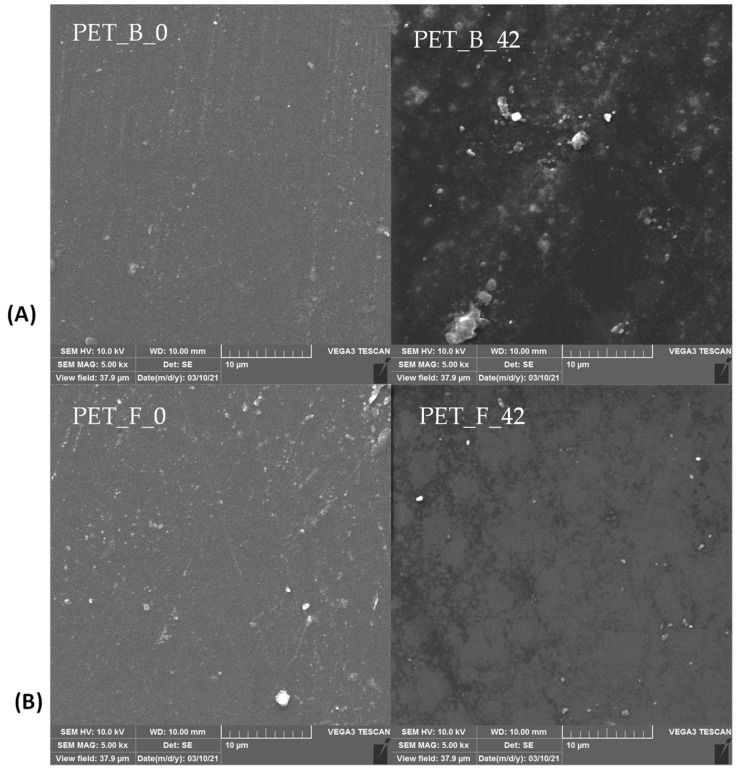
SEM micrographs of (**A**) PET bottle and (**B**) PET foil samples; magnification was 5000×.

Scanning electron microscopy (SEM) was used to analyze the surface morphology of the studied PET samples, and the microscopic images are shown in Figure 1. Images of both PET samples show a smooth surface without cracks, fractures, notches, and bumps characteristic of photooxidative decomposition. It is well known [43] that in thermooxidative aging, the polymer ages throughout its mass, while UV aging affects only the surface. In other words, in thermooxidative aging, the mass of the material ages more than the surface, and because of the low degradation kinetics, the defects on the surface are less visible, as can be seen in the microscopic images of SEM. After aging for 42 days, some irregularities in the structure can be observed, and more white spots and defects are visible in the material. This is believed to be the result of the restructuring of the crystalline and amorphous phases in the polymer.

### 3.5. Particles Size Distribution

**Figure 2:**
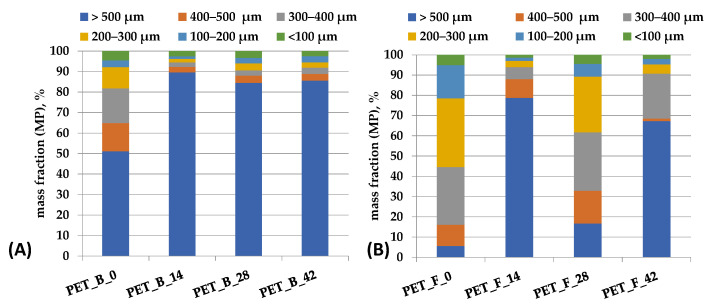
Fractions of particle size distribution of pristine and aging (**A**) PET bottle and (**B**) PET foil samples after grinding and sieving.

After thermooxidative aging, PET samples were ground in a cryogenic mill to prepare MP samples for further study. The particles of MPs were sieved, and the particle size distribution is shown in the mass % per fraction (Figure 2). It is evident that the particle size distribution of the pristine PET bottles and foils is different from that of the thermooxidatively aged samples, indicating changes in the polymer structure. For the PET bottles, the percentage of particle size >500 μm is very high (51%) and increases with thermooxidative aging to 86% after 42 days (PET_B_42, Figure 2), indicating an increase in toughness and a decrease in brittleness. It is concluded that this is the result of an increase in the amorphous phase in the samples, which is also evident for the PET foils after 42 days of aging, where the fraction of particle size >500 μm increased significantly compared to the pristine sample (PET_F_0) by up to 67% (PET_F_42). The fragmentation results obtained are in agreement with the DCS analyses, which showed that the fraction of the crystalline phase decreases with aging, as a result of degradation, i.e., due to the cleavage of the polymer macromolecules. The fractions of the particle size distribution in the range of 100–500 μm for foils are higher due to the initial ratio of amorphous to crystalline phase, indicating a higher fraction of crystalline phase in the pristine sample [44,45].

### 3.6. Influence of Key Parameters on Adsorption of DCF on Pristine and Aged MPs

RSM was applied to study the influence of operating parameters such as the temperature and dosage of MPs on DCF adsorption. A combined experimental and statistical/modeling approach was used to account for the possible interactions. The chosen experimental plan was FFD. Therefore, these process parameters are represented by independent variables and translated into dimensionless coded values—*X*_1_ (*T*, °C) and *X*_2_ (*γ*(MP), mg L^−1^)—at the levels shown in Table S2 with the complete experimental design matrix. The adsorption coefficient (*K*, Lg^−1^) is commonly used to describe the affinity of an adsorbate for an adsorbent and is used to predict the performance of adsorption processes [46,47,48], so it was used in our study as the response (*Y*) for the adsorption process (the calculations are detailed in Text S4).

Their combined influences on the adsorption process are described by polynomial equations (Text S4, Equations (S1)–(S4)) and a three-dimensional contour plot (Figure S7) together with the diagnostic analysis of the models (Figure S6).

The equilibrium concentration of DCF in the aqueous solution was achieved within 24 h. For both bottle- and foil-originated MPs, adsorption was higher in aged material. The experimental conditions yielding the highest DCF adsorption were obtained by pristine and aged PET_B and PET_F, as well as the adsorption extents obtained and the corresponding calculated and predicted *K* values, which are shown in Table 5. The calculated values of the *K* coefficient based on the experimental results are in good agreement with the predicted values. It can be noticed that adsorption generally favors low dosages of MP and lower temperatures.

According to the analysis of variance (ANOVA), *F* and *p* tests were performed to check the influence of the studied parameters and their combination on the degree of adsorption and the validity of both models (Tables S3 and S4). The significance of individual model term *X*_1_ (*T*, °C) was high (*p* < 0.05, high *F* value) in the case of pristine MPs (M1, M3), which means that the adsorption is affected by the changes in the temperature of the system (Table S3). On the other hand, *X*_2_ (*γ*(MP), mg L^−1^) has a lower *F* value, from which it can be concluded that the dosage of MPs is less relevant, but at the same time not an ignorable parameter. This is also confirmed by a high *F* value for the model term that also represents the temperature of the system (*X*_1_^2^) and a low *F* value for the interaction model term *X*_1_ × *X*_2_, which combines the effect of temperature and concentration of MPs.

According to the RSM surface plot (Figure S7), it appears that adsorption prefers a lower *T* in the case of both pristine and aged MPs (bottles and foils), which can be explained by the fact that the molecular kinetic energy and temperature are proportional units. A lower temperature (e.g., 6.25–11.5 °C, Table 5) would result in slow molecular motion, which decreases desorption, i.e., DCF molecules would remain adsorbed on the surface. Changing the dosage of MPs showed no significant effect for both pristine and aged MPs, while adsorption favored a lower dosage of MPs in all cases (250 mg L^−1^). This may be related to the fact that aged foils have the highest content of small particles, which was already explained in the size distribution referred to in the discussion above. Smaller MP particles have a higher specific surface area, which is important for the adsorption process, and this would imply that less MPs are needed to achieve the same results as in the case of pristine foils [49].

The pH of the aqueous DCF solution, which was slightly acidic (~6), was adjusted to a neutral value, i.e., pH 7. According to the literature, acidic pH values are more favorable for the adsorption of inorganic pollutants (e.g., metal ions) on the hydrophobic MP surfaces, while increasing the pH towards neutral (7) would result in lower adsorption as the desorption process would be initiated [50]. In the case of the adsorption of hydrophobic organic pollutants (such as DCF) on hydrophobic MPs (such as PET, PE, and PP), the maximum adsorption is observed to be around pH 7, while at higher values, desorption takes on the main role [51]. The electrostatic interactions between pollutant and MPs depend on a specific pH of the solution in which the MPs are present, as well as on the point of zero charge (pH_PZC_) of the MPs themselves and on the pKa of the pollutant.

When the pH of the adsorption environment exceeds the pH_pzc_ of the MPs, their surface is negatively charged and can electrostatically attract positively charged organic pollutants. The p*K*_a_ value of DCF is 4.15 (Table S5), which means that DCF (as a weak acid) is in the deprotonated form at pH 7. The determined values of the pH_pzc_ of pristine MPs were 2.6 and 2.8 for PET_B_0 and PET_F_0, respectively (Figure S5). It should be noted that the determination of pH_pzc_ for the aged MPs was not possible. Electrostatic interactions between deprotonated DCF and negatively charged pristine MPs are less likely because both are negatively charged, which leads to electrostatic repulsion and inhibits adsorption [52]. Such an effect can plausibly explain the rather low adsorption of DCF on pristine MPs in our case. The slightly higher adsorption on aged MPs can be related to surface deterioration rather than to the significant changes in pH_PZC_ values between the pristine and aged samples. The highest adsorption of DCF was found in the aged foils (7.2%, Table 5), which was expected since PET_F_42 is a more hydrophobic MP with a high percentage of small particles (30% of the 100–400 µm fraction) and a higher C.I. value, i.e., a higher content of carbonyl and carboxyl groups, compared to its pristine analogue. A similar case was reported in the study by Liang et al. [53], which demonstrated the adsorption of DCF on UV-aged poly(butylene adipateco-terephthalate)—PBAT. The particle size of PBAT ranged from 75 to 150 µm, while aged samples showed a decrease in crystallinity and an increase in the oxygen-containing group, as well as a decrease in hydrophobicity. Therefore, they suggested that the interactions were hydrophobic interactions, focusing on the hydrogen–halogen bonds formed between the nitrogen, oxygen, and chlorine atoms on DCF and the hydrogen atoms on MPs. Additionally, electrostatic interactions were suggested due to the surface charge of PBAT MPs and the ionic form of DCF at the optimal acidic pH conditions in their case, as well as π–π stacking due to the fact that both DCF and PBAT MPs have and share an affinity with aromatic rings [53]. Accordingly, the analogy with our study can be drawn to some extent. The adsorption of DCF on aged MPs may be facilitated by hydrophobic interactions, hydrogen bonding, and π–π stacking as a result of surface modifications during the aging process. The electrostatic interactions are unlikely for the pristine PET MPs due to the repulsion between the negatively charged surface and the deprotonated DCF molecule. However, the same cannot be said with certainty for the aged samples since it was not possible to determine the pH_pzc_, but as mentioned earlier, it is more likely that adsorption occurred due to surface changes as a result of the aging process. Therefore, it is important to note that the least crystalline plastics should be able to accumulate the highest amount of organic pollutants, in contrast to the amorphous fractions, which are rigid and flexible [54]. As mentioned above, the aging process led to an increase in the amorphous phase of PET_B_42 and PET_F_42 compared to PET_B_0 and PET_F_0, respectively, which is in favor of the results obtained in the adsorption tests. Furthermore, FTIR analysis (Table 2) confirmed that the oxygen-containing functional groups of PET increased after thermo-oxidative aging. In addition, a 16% increase in carbonyl indices was recorded for PET foils compared to the sample prepared from bottles. The literature suggests that the presence of such functional groups on the surface of aged MPs can increase the polarity as well as the hydrophobicity of the material, which favors the adsorption of hydrophobic organic contaminants. Li et al. showed that decreasing the size of polystyrene MPs (75–215 µm) increases the adsorption of the hydrophobic (log*K*_ow_ = 4.76) antibacterial agent triclosan [55]. Accordingly, the adsorption of hydrophobic DCF (log*K*_ow_ = 4.51) was also expected in our study.

Based on the FTIR results, we can reasonably conclude that the proton donor functional groups present in aged PET_B and PET_F (carbonyl group, carboxyl group) are involved in the adsorption of DCF molecules (amino group) via hydrogen bonding. This can increase the overall adsorption affinity, which was also discussed by Wang et al. [56].

Although the contact angle of pristine PET bottles and foils is <90°, indicating that PET is generally a hydrophilic material, the contact angle was found to increase with aging increasing the possibility of adsorption.

The adsorption of DCF in our study followed an increasing order PET_B_0 < PET_F_0 < PET_B_42 < PET_F_42; the values obtained for the maximum amount of adsorbed DCF are summarized in Table 5. These results indicate that DCF adsorption increases moderately with the aging of PET.

### 3.7. Aquatic Toxicity Assessment

**Figure 3:**
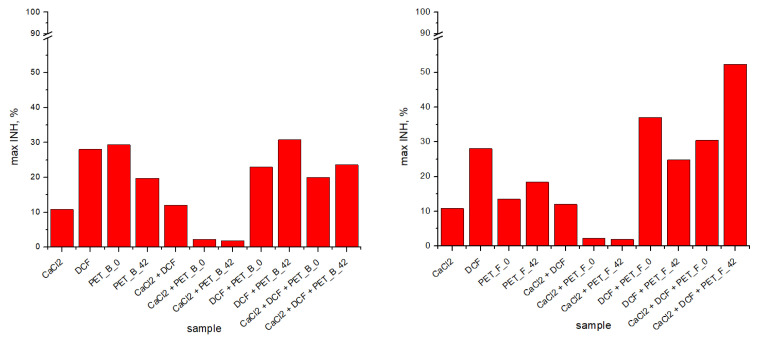
Inhibition of samples PET_B (**left**) and PET_F (**right**) was determined after 72 h of exposure to *Pseudokirchneriella s.*; inhibition refers to the undiluted samples.

Aquatic toxicity was tested both on individual MPs and in combination with DCF (after the adsorption process was conducted). The results were expressed as a maximum inhibition of the tested algae *Pseudokirchneriella subcapitata*, which is a measure of the maximum toxicity (Figure 3) of the tested samples.

**Figure 4:**
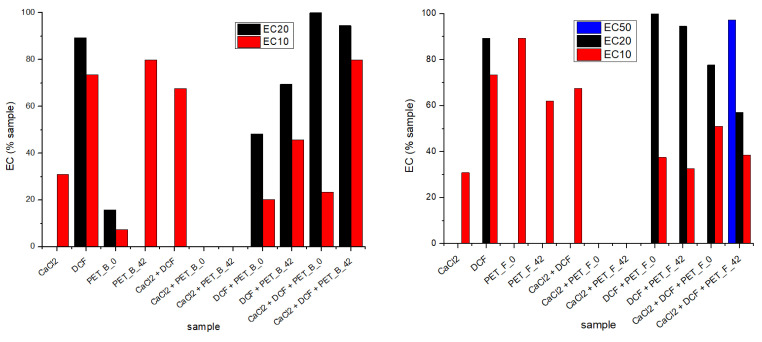
Effective concentrations of pristine and aged PET_B (**left**) and PET_F (**right**) were determined after 72 h of exposure to *Pseudokirchneriella s*.

Only for the aged foils was the inhibition of algal growth greater than 50% after 72 h of exposure to the microalgae population (Figure 4). To determine the total toxic impact that each component could cause (PET, DCF, and CaCl_2_), the toxicity of each individual component was separately investigated and compared.

The inhibition of algal growth was used to calculate the effective amount of the sample that causes 10%, 20%, and 50% inhibition of the species tested (EC_10_, EC_20_, EC_50_), as represented in Figure 4, and explained in Text S2.

Individually tested DCF caused 28% inhibition of microalgae at 0.054 mmol dm^−3^ (50 µM), which was the highest concentration tested and also very close to the concentration of DCF in our case. It must be noted that the investigation of DCF toxicity on microalgae *Pseudokirchneriella subcapitata* was previously studied by Quinn et al. [57], who reported that 50% inhibition was detected at around 0.22 mmol dm^−3^, which was a concentration four times higher than in our case.

In the case CaCl_2_ alone, the inhibition was about 10%, although the initial concentration was relatively high compared with the other components (0.01 mmol dm^−3^). A similar result was reported by Simmons et al. [58], where a 10%effective concentration was measured at around 0.0026 mmol dm^−3^. Husseini et al. [59] reported a positive effect of high CaCl_2_ concentrations (0.005–0.01 mmol dm^−3^) on the growth of the microalga *Chlorella vulgaris* despite gamma irradiation and claimed that chloride plays a central role in many defense mechanisms triggered by abiotic stressors. In our case, the effect of added CaCl_2_ was similarly positive. It can be noticed that CaCl_2_ must play a role in the overall toxic effect in complex samples containing MPs and DCF, since the inhibition did not increase but remained more or less the same as in the case of individual MPs samples. The same effect was observed with CaCl_2_ and MPs and with the combination of all three components (Figure 4).

The combined toxicities of PET MPs and DCF were slightly altered compared with the individual components. Since MP can be a vector for different organic pollutants, it is necessary to investigate the synergistic, antagonistic, and additive toxicity effects caused by the presence of MPs in DCF solution. In such complex samples, the MPs can cause an increase in toxic effects on the one hand but also enhance stimulation of the algal growth on the other hand. In the study by Heinlaan et al. [60], the toxicity of polystyrene nanoplastic was tested on *Pseudokirchenriella s.*, *D. magna*, and *V. fischeri*, and none of the organisms showed inhibition or mortality. In contrast, in the case of Li et al. [61], MPs reduced the toxicity of the pharmaceutical sulfamethoxazole to the marine algae *Skeletona costatum* compared to the toxicity caused by a sole pharmaceutical. Higher adsorption capacity alleviated the toxicity of MPs by increasing the hydrophobicity of MPs and enhancing the aggregation of MPs, which might reduce the contact with the algal population. This is a clear example of the antagonistic effect that MPs can cause in complex systems. A similar effect may have occurred in our case as well.

## 4. Conclusions

The study investigated the correlation between aging of PET MPs and the changes in physicochemical properties and size distribution influencing the adsorption capacity of DCF, and the resulting effects on the combined toxicity to the freshwater algae *Pseudokirchneriella subcapitata*. Aging resulted in mild changes in surface morphology, while an increase in hydrophobicity was observed in both PET bottles and foils. Aging significantly affected the size distribution, with large fractions such as >500 μm increasing in the aged samples compared to pristine ones. As a consequence of changes in physicochemical properties, aged samples exhibited a higher adsorption capacity for DCF than pristine materials. These results can be attributed to the slightly increased hydrophobicity and decreased crystallinity upon aging, which enable the adsorption of DCF via hydrophobic interactions and π–π stacking. The adsorption tests revealed that the temperature was more influential than the MP dosage. However, both pristine and aged PET MPs favored DCF adsorption at lower temperatures and lower dosages of MPs. The toxicity tests showed that both MPs and DCF contributed to the overall toxicity. The synergistic effect can be observed in the case of a ternary mixture for PET_F_42, while PET_B_42, PET_B_0, and PET_F_0 exhibited antagonistic effects. On the other hand, CaCl_2_ showed antagonistic effects in both binary and ternary mixtures, mitigating individual toxic effects caused by MPs and/or DCF, except in the case of the ternary mixture with PET_F_42, where synergistic effects can be observed. Thus, it can be concluded that aged PET MPs may adsorb pollutants more readily and serve as a vector for their further transport, while also having increased toxic effects on freshwater species.

## Figures and Tables

**Table 1:** Experimental range and levels of investigated parameters in the process of adsorption.

Parameters	Model Variables/Coded Values	Level/Range		
		−1	0	1
*T*, °C	*X*_1_	5	20	35
*γ* (MP), g L^−1^	*X*_2_	250	500	750

**Table 2:** The absorption ratio of carbonyl, oxyethylene groups, *gauche* and *trans* conformation of PET bottle and foils.

Sample	$A=(\frac{1711}{1404})$	$A=(\frac{1233}{1404})$	$A=(\frac{1376}{1404})$	$A=(\frac{1343}{1404})$	$A=(\frac{972}{1404})$	$A=(\frac{1090}{1404})$
	C=O	C-O	*gauche*	*trans*	*gauche*	*trans*
PET_B_0	6.73	9.36	0.27	1.16	1.61	4.79
PET_B_14	6.87	10.56	0.30	1.16	1.92	5.24
PET_B_28	7.32	12.77	0.30	1.18	2.11	5.05
PET_B_42	7.01	11.56	0.32	1.05	1.45	4.36
PET_F_0	6.77	12.55	0.44	0.32	0.76	6.69
PET_F_14	7.20	14.69	0.43	0.32	0.77	7.16
PET_F_28	6.78	15.08	0.45	0.32	1.03	6.52
PET_F_42	8.53	12.11	0.43	0.32	0.86	6.33

**Table 3:** DSC results of PET bottle and foils; endothermic (Δ*H*_m_), exothermic transition (Δ*H*_c_), and temperatures of melting (*T*_m_), crystallinity (*T*_c_), glass transition (*T*_g_), and degree of crystallinity (*X*).

	PET_B_0	PET_B_14	PET_B_28	PET_B_42
***T*_m_ (°C)**	248	248	248	247
***T*_g_ (°C)**	80	80	80	80
***T*_c_ (°C)**	183	156	156	157
***T*_cc_ (°C)**	/	148	148	147
**Δ*H*_m_ (J g^−1^)**	35	34	33	33
**Δ*H*_c_ (J g^−1^)**	37	25	27	23
**Δ*H*_cc_ (J g^−1^)**	/	1.7	2.5	0.6
***X* (%)**	25.6	22.7	21.4	22.8
	**PET_F_0**	**PET_F_14**	**PET_F_28**	**PET_F_42**
***T*_m_ (°C)**	248	250	249	249
***T*_g_ (°C)**	80	81	80	80
***T*_c_ (°C)**	177	176	176	176
**Δ*H*_m_ (J g^−1^)**	35	29	31	29
**Δ*H*_c_ (J g^−1^)**	39	33	36	34
**Δ*H*_cc_ (J g^−1^)**	1.03	1.03	1.03	1.03
***X* (%)**	25.7	20.9	22.1	20.8

**Table 4:** Contact angle values (*θ*) for the samples of PET bottle (PET B) and PET foil (PET F) in contact with water, aged for: 0, 14, 28, and 42 days.

Samples	PET_B_0	PET_B_14	PET_B_28	PET_B_42
*θ* (°)/water	69	79	76	76
**Samples**	**PET_F_0**	**PET_F_14**	**PET_F_28**	**PET_F_42**
*θ* (°)/water	61	68	67	72

**Table 5:** Experimental conditions and responses for adsorption expressed via *K* coefficient in the case of the pristine and aged MP bottles and foils.

	Parameter		Experimental		Response	Model
Sample	*T*, °C	*γ*(MP), mg L^−1^	DCF Adsorption			
			Maximal, %	*K*, L g^−1^	*K*, L g^−1^	
PET_B_0	11.50	250	5.00	0.230	0.210	M1
PET_B_42	6.25	250	6.11	0.262	0.260	M2
PET_F_0	8.50	250	5.28	0.236	0.223	M3
PET_F_42	8.00	250	7.22	0.340	0.249	M4

## Data Availability

The data presented in this study are available upon reasonable request from the corresponding author.

## Supplementary Materials

The following supporting information can be downloaded at: https://www.mdpi.com/article/10.3390/toxics11070615/s1, Figure S1: Fundamental chain scission of thermal process in PET; Figure S2: Low-molecular-weight volatile products of PET degradation; Figure S3: Production of acetaldehyde from vinyl ester; Figure S4: FTIR spectra of PET: (a) bottle and (b) foil samples aged: 0, 14, 28 and 42 days; Figure S5: Zeta potential of the PET B 0 and PET B 42 sample; Figure S6: Validation of the models **M1**–**M4** ((a–d), respectively): diagnostic analysis of the residue (left) and Box-cox analysis (right); Figure S7: Three-dimensional response surface and contour diagrams showing the effects of the mutual interactions of initial pH and γ(PET-MP) (left), T and γ(PET-MP) (middle) and initial pH and T (right) on the response (adsorption of DCF); Table S1: Explanation of the sample abbreviations for the toxicity bioassays; Table S2: Experimental design matrix with two independent variables for each process expressed in coded units and actual values for models **M1**, **M2**, **M3**, and **M4**; values observed during the treatments and predicted by **M1**, **M2**, **M3**, and **M4**, respectively; Table S3: Analysis of variance (ANOVA) of the response surface models **M1**–**M4** predicting adsorption of DCF on PET MPs, respectively; Table S4: Statistical analysis of regression coefficients for models **M1**–**M4**; Table S5: Physicochemical properties and molecular structure of PET and DCF. References [62,63,64,65] are cited in the Supplementary Materials.
