# Supplementary material for: Effect of Aging on Physicochemical Properties and Size Distribution of PET Microplastic: Influence on Adsorption of Diclofenac and Toxicity Assessment
Source: Toxics. 2023 Jul 14;11(7):615. doi: 10.3390/toxics11070615 (PMC10383551; doi:10.3390/toxics11070615)
Supplement: Supplementary file 1 [file toxics-11-00615-s001.zip › toxics-2439679-supplementary.pdf]

toxics

Supplementary Materials

Figures S1–S3 were drawn in the software ChemDraw Professional 15.0, PerkinElmer, USA.

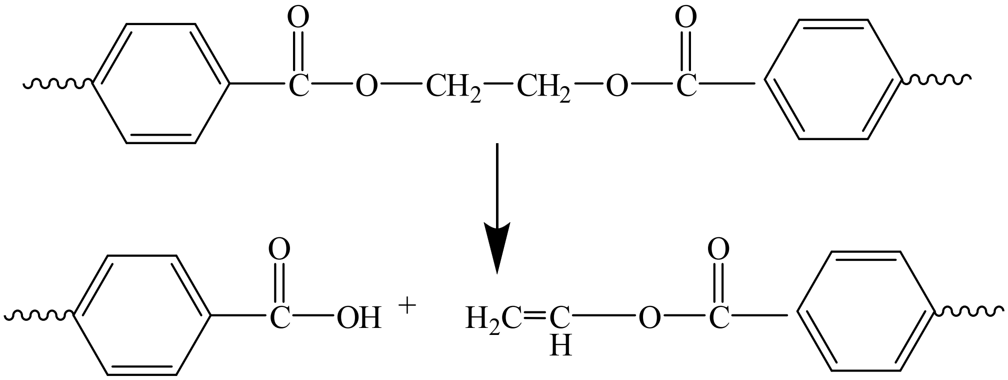

Figure S1. Fundamental chain scission of thermal process in PET.

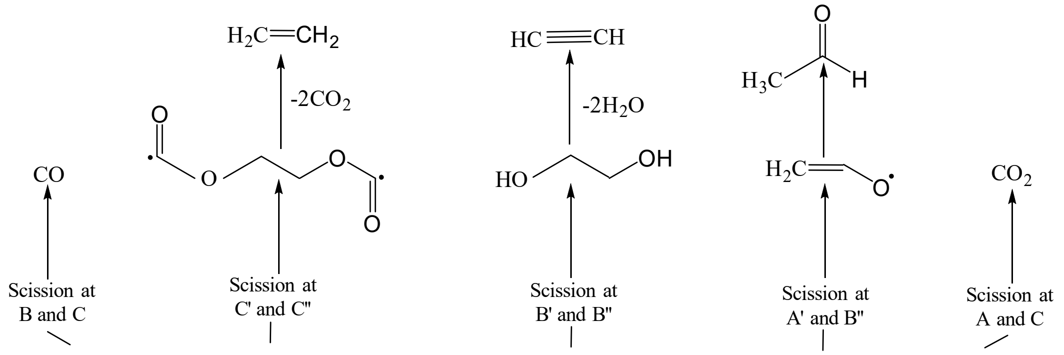

Figure S2. Low molecular volatile products of PET degradation.

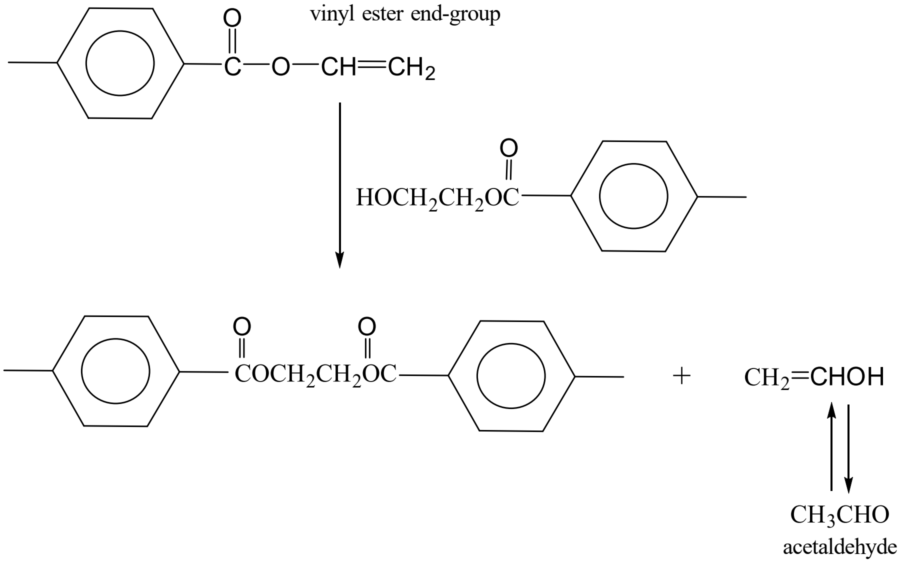

Figure S3. Production of acetaldehyde from vinyl ester.

Text S1 Detailed information of the characterization analysis of PET samples

The effects of aging of PET plastic films were investigated by ATR-FTIR spectroscopy using a Specrum One (Perkin Elmer, USA) spectrometer. The spectra were recorded in the range from 4000 cm^{-1} to 650 cm^{-1} with a spectral resolution of 4 cm^{-1}, in average of four scans. As an indicator of degradation and chemical changes occurred during thermooxidative aging, the indices of carbon-oxygen bonds (C–O) and carbonyl group bonds (C=O) were followed and carbonyl index (C.I.) was calculated. The C.I. was calculated from the ratio between the integrated band absorbance of the carbonyl (C=O) peak from 1850 to 1650 cm^{-1} and that of the methylene (CH_2) scissoring peak from 1500 to 1400 cm^{-1} as expressed in the following Equation (1):

$$\mathrm{C.I.}\,(\mathrm{C=O}) = \frac{\text{Area under band } A_{1850-1650\ \mathrm{cm}^{-1}}}{\text{Area under band } A_{1500-1400\ \mathrm{cm}^{-1}}} \quad (1)$$

Copyright: © 2023 by the authors. Licensee MDPI, Basel, Switzerland. This article is an open access article distributed under the terms and conditions of the Creative Commons Attribution (CC BY) license (https://creativecommons.org/licenses/by/4.0/).

For the carbon-oxygen bonds (C-O), peak from 1300 to 1140 cm^{-1} were taken and brought into correlation with that of methylene (CH_2) scissoring peak from 1500 to 1400 cm^{-1}, as expressed by the following Equation (2):[2]

$$\mathrm{C.I.}\,(\mathrm{C–O}) = \frac{\text{Area under band } A_{1300-1140\ \mathrm{cm}^{-1}}}{\text{Area under band } A_{1500-1400\mathrm{cm}^{-1}}} \quad (2)$$

The area under the band is calculated through the Perkin Elmer instrument software options using the peak analysis tool.

The surface wettability was evaluated through water contact angle measurements for five replicates. These measurements were conducted using a Data Physics OCA20 goniometer and measurements were performed at room temperature. The sessile drop method was used to determine the contact angle on pristine and aged PET. The drop volume was 2 μL.

Further characterization of the PET samples was performed on a differential scanning calorimeter, Mettler Toledo DSC 823eT in 2 cycles. Two heating cycles were conducted from 25 °C to 280 °C, while THE cooling cycle was conducted back to 25 °C, at a heating/cooling rate of 10 °C / min. Thermogravimetric analysis (TGA) was performed on Q500 analyzer (TA Instruments, USA). The measurements were carried out non-isothermally, in the temperature range from room temperature to 600 °C, heating rate was 10 °C min^{-1} in nitrogen with a flow rate of 60 ml min^{-1}. The water absorption uptake was determined by TGA, heating from 25 to 120 °C for 2 hours at rate of 20 °C /min in nitrogen with a flow rate of 60 ml/min. Before the measurement all samples have been exposed to moisture in a laboratory oven at 100 °C for one hour together with heated water in 2 L glass crystallizing dish.

The microstructural morphologies of the pristine and aged samples were studied using a Vega III scanning electron microscope (SEM, Tescan, Czech Republic), with the filament operated at 10 kV. To achieve conductivity of the sample surface, a thin layer of gold/palladium (60:40) was sputtered onto the films under high vacuum using an sputter coater (EMS 550X, UK).

Text S2 Detailed procedures for the aquatic toxicity determination and calculations

To determine the influence of the MPs particles on the total optical density of the samples, the initial optical density in each test cuvette was measured and taken into account later in the calculations of growth rate (Table S1). All measurements were conducted in triplicates and standard deviation was calculated. The average growth rate in the control replicates were all above minimal 1.4 day^{-1}. Inhibition was measured as a reduction in

specific growth rate (μ_i) relative to control cultures grown under identical conditions. The optical density for each test and control batch replicate (long-cell cuvettes) was correlated with the cell density and used for the calculation of the specific growth rate (μ_i, day^{-1}), using Equation (3):

$$\mu_i = \frac{\ln n_L - \ln n_0}{t_L - t_0} \tag{3}$$

where n_0 an n_L are the initial and measured cell density in time, while t_0 and t_L are times of the test start and last measurement. The inhibition (I, %) was determined according to the Equation (4):

$$I_\mu = \frac{\mu_c - \mu_0}{\mu_c} \times 100 \tag{4}$$

where the μ_C and μ_0 are growth rate for the test and control batches.

The Effective Concentration (EC) was then determined using OriginPro 8.5. software employing the dose-response function. In this work, samples were complex, therefore EC for each sample was defined not through concentrations, but as a % of the sample in each dilution step, according to the geometric series with the ratios sample – culturing algal medium from 1:1 to 1:16.

Table S1. Explanation of the sample abbreviations for the toxicity bioassays.

#	Label	c (DCF), µM	c ($CaCl_2$), mM	γ (MPs), g	V (sample), mL
**1**	DCF	50	0	0	100
**2**	$CaCl_2$	0	0.01	0	100
**3**	PET_B_0	0	0	250	100
**4**	PET_B_42	0	0	250	100
**5**	PET_F_0	0	0	250	100
**6**	PET_F_42	0	0	250	100
**7**	$CaCl_2$ + DCF	50	0.01	0	100
**8**	$CaCl_2$ + PET_B_0	0	0.01	250	100
**9**	$CaCl_2$ + PET_B_42	0	0.01	250	100
**10**	$CaCl_2$ + PET_F_0	0	0.01	250	100
**11**	$CaCl_2$ + PET_F_42	0	0.01	250	100
**12**	DCF + PET_B_0	50	0	250	100
**13**	DCF + PET_B_42	50	0	250	100
**14**	DCF + PET_F_0	50	0	250	100
**15**	DCF + PET_F_42	50	0	250	100
**16**	$CaCl_2$ + DCF + PET_B_0	50	0.01	250	100
**17**	$CaCl_2$ + DCF + PET_B_42	50	0.01	250	100
**18**	$CaCl_2$ + DCF + PET_F_0	50	0.01	250	100
**19**	$CaCl_2$ + DCF + PET_F_42	50	0.01	250	100

Text S3 Characterization results for pristine and aged PET

FTIR spectra for both pristine and aged bottles and foils are given in Figure S4.

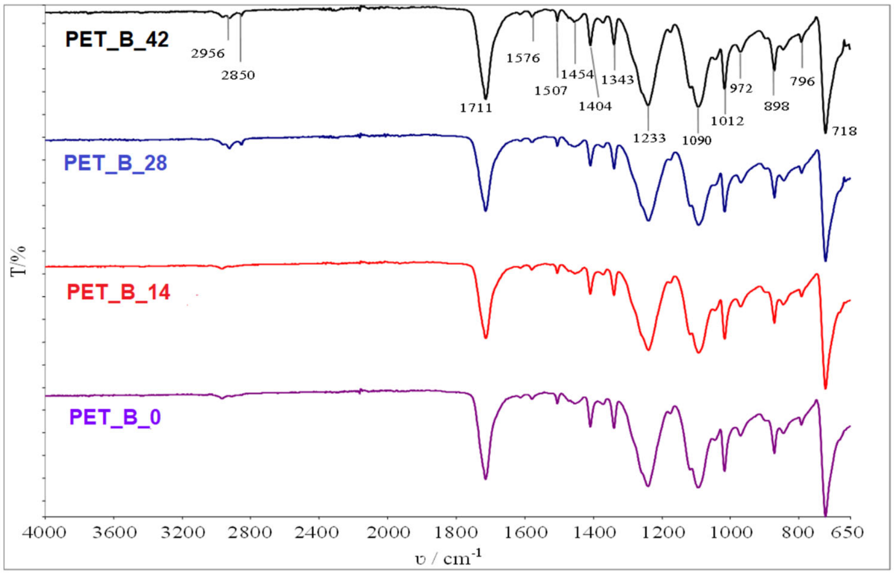

(a)

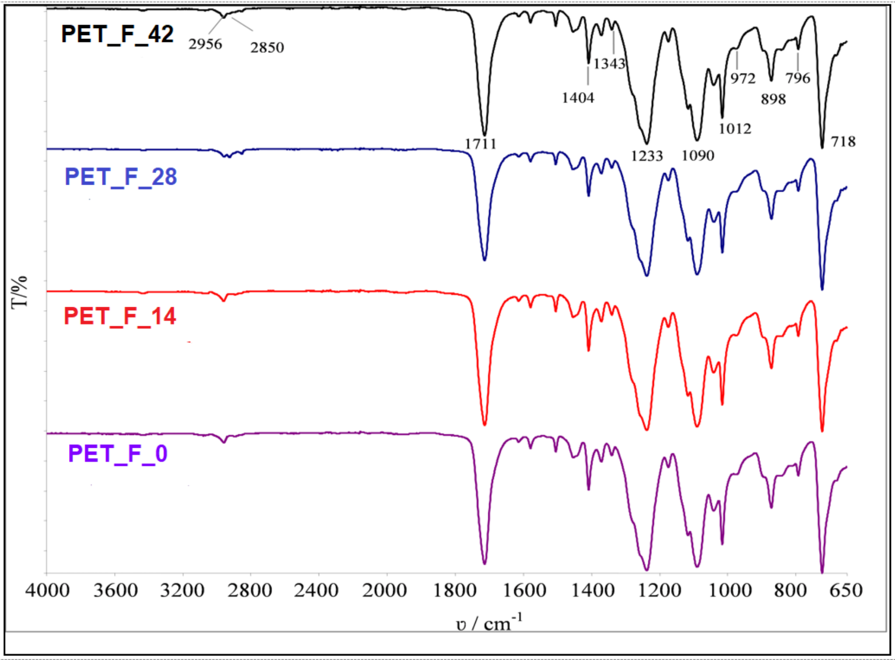

(b)

Figure S4. FTIR spectra of PET: (**a**) bottles and (**b**) foils samples aged: 0, 14, 28 and 42 days.

Figure S5 shows the zeta potential, indicating the instability of the PET particles (negative zeta potential). Besides, we were not able to determine the pH_{pzc}, which means that the PET particles themselves do not have a pronounced charge. It can be noticed that pH_{pzc} for aged MP can not be expressed.

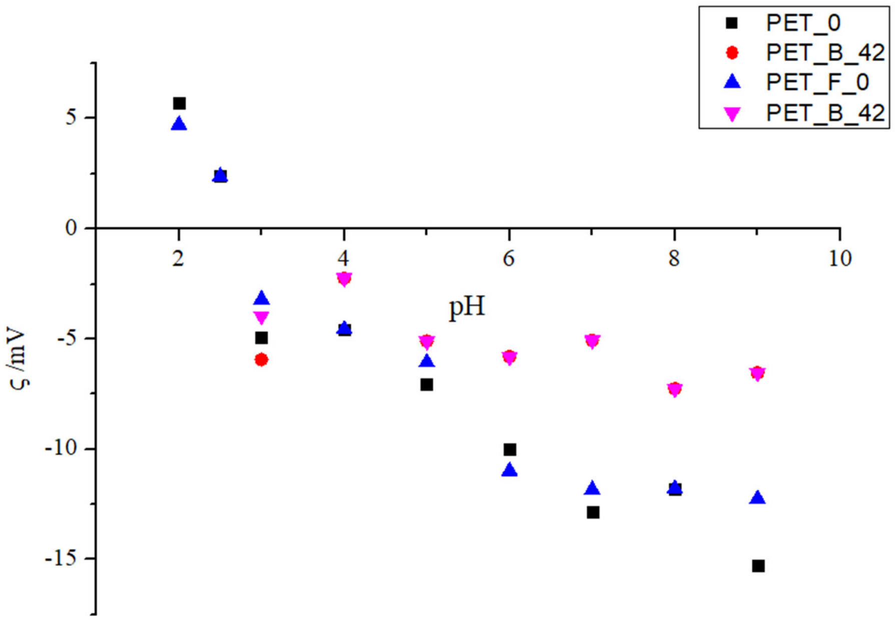

Figure S5. Zeta potential of the PET B 0 and PET B 42 sample.

Text S4 Results for response surface methodology calculations

The adsorption coefficient[3,4] (K, Lg^{-1}) was calculated according to the Eq. (5):

$$K_C = \frac{q_e}{c_e} \quad (5)$$

where c_e is the equilibrium concentration (mg L^{-1}) of pollutants in the aqueous phase and q_e (mg g^{-1}) is the amount of adsorbed organics on the PET-MP at equilibrium, which is calculated according to the Eq (6):

$$q_e = \frac{c_0 - c_e}{c_a} \quad (6)$$

where c_0 (mg L^{-1}) is the average concentration of organic compound in the initial samples, c_e (mg L^{-1}) is the average concentration of organic compound in the solution at the equilibrium; and c_a (mg L^{-1}) is the concentration of adsorbent.

Their combined influences on processes effectiveness, which were expressed by the means of DCF adsorption level (i.e. dependent variable; Y) for adsorption processes are described by polynomial equations (1-4) (Table S2), respectively (Eq 7-10):

$$\mathbf{M1} \quad Y_1 = 0.185 - 0.076X_1 - 0.020X_2 + 0.005X_1X_2 - 0.075X_1^2 + 0.003X_2^2 \quad (7)$$

$$\mathbf{M2} \quad Y_1 = 0.201 - 0.105X_1 - 0.013X_2 + 0.012X_1X_2 - 0.077X_1^2 + 0.004X_2^2 \quad (8)$$

$$\mathbf{M3} \quad Y_1 = 0.177 - 0.097X_1 - 0.014X_2 + 0.009X_1X_2 - 0.081X_1^2 + 0.010X_2^2 \quad (9)$$

$$\boldsymbol{M4} \quad Y_1 = 0.277 - 0.091X_1 - 0.015X_2 + 0.018X_1X_2 - 0.08X_1^2 + 0.017X_2^2 \quad (10)$$

Calculations and analyses were performed using Design Expert 10.1 (StatEase, USA) and STATISTICA v.14 (TIBCO, USA). Statistical analysis of the significance of obtained data was performed according to the ANOVA, in terms of F and p test.

Table S2. Experimental design matrix with two independent variables for each process expressed in coded units and actual values for models M1, M2, M3, M4; values observed during the treatments and predicted by M1, M2, M3, M4, respectively.

**Variables**				Response (K, L g^{-1})			
X_1		X_2		Y			
**coded**	***T*, °C**	**coded**	γ(MP), g L^{-1}	PET_B_0	PET_B_42	PET_F_0	PET_F_42
-1	5	-1	250	0.211	0.260	0.223	0.235
0	20	-1	250	0.211	0.211	0.211	0.299
1	35	-1	250	0.047	0.030	0.008	0.012
-1	5	0	500	0.182	0.236	0.195	0.182
0	20	0	500	0.188	0.195	0.169	0.264
1	35	0	500	0.036	0.019	0.004	0.006
-1	5	1	750	0.167	0.200	0.181	0.154
0	20	1	750	0.157	0.205	0.171	0.299
1	35	1	750	0.023	0.017	0.003	0.004

The fitting of models was evaluated using the coefficient of determination (R^2) and the analysis of variance (ANOVA), results are summarized in Table S3 and Table S4**.**

The full ANOVA results, including statistical parameters for models M1-M4, are summarized in Table S3. Table S4 represents a statistical analysis of regression coefficients for models M1-M4. ANOVA revealed that models M1-M4 are accurate ($0.9774<R^2<0.9967$) and significant ($0.0001<p<0.1501$).

Table S3. Analysis of variance (ANOVA) of the response surface models M1 – M4 predicting adsorption of DCF on pristine and aged MP, respectively.

**Parameter**		**Model**	X_1 (***T*, °C**)	X_2 (**γ (MP), mg L^{-1}**)	X_1X_2	X_1^2	X_2^2
***F*-value**	M1	169.8472	606.9024	43.0111	1.7763	197.5426	0.0039
	M2	110.4771	459.9671	7.0438	3.9252	81.2596	0.1896
	M3	182.3132	721.3548	15.8948	4.2359	167.4740	2.6066
	M4	70.3035	141.8488	3.7005	3.7616	200.6535	1.5530
***p*-value**	M1	0.0007	0.0001	0.0072	0.2748	0.0008	0.9541
	M2	0.0013	0.0002	0.0767	0.1419	0.0029	0.6926
	M3	0.0006	0.0001	0.0283	0.1317	0.0010	0.2048
	M4	0.0026	0.0013	0.1501	0.1478	0.0008	0.3011

*$p < 0.05$ is considered as significant

Table S4. Statistical analysis of regression coefficients for models M1-M4.

Parameter	Model			
	**M1**	**M2**	**M3**	**M4**
R^2	0.9965	0.9946	0.9967	0.9915
**Adj-R^2**	0.9906	0.9856	0.9913	0.9774

Diagnostic analysis (RD) for the developed RSM models **M1**, **M2**, **M3** and **M4** is performed, including a normal probability test which revealed that errors are normally distributed and independent of each other (Figure S6, top row). In the case when the ratio of highest and lowest response is bigger than 10, there is a possibility that transformation would increase model accuracy. Hence, Figure S6 (bottom row) represents a Box-Cox graph; since in all cases (M1-M4) ratios of minimal and maximal responses were bigger than 10, a natural logarithm of response was applied.

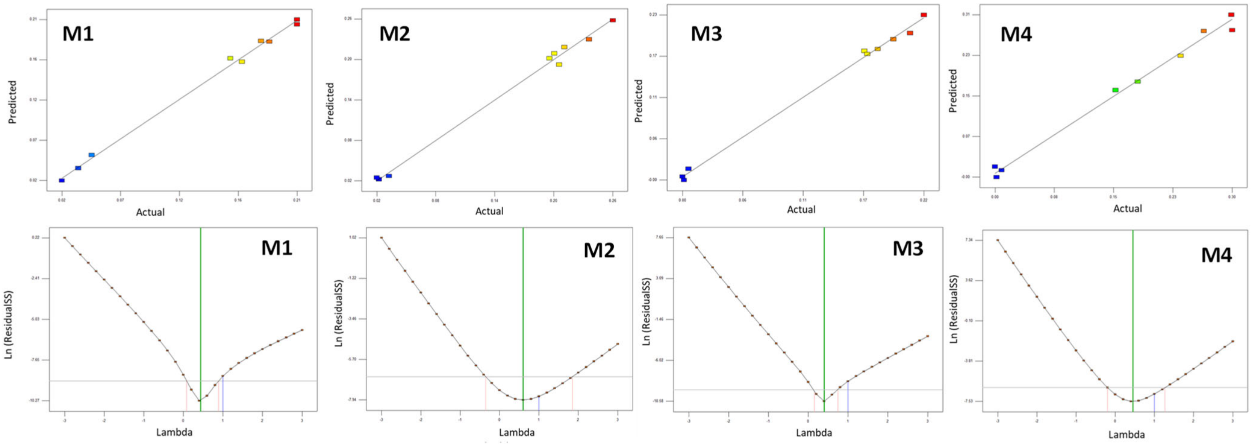

Figure S6. Validation of the models M1-M4: a diagnostic analysis of the residue (top row) and Box-cox analysis (bottom row).

Combined impact of process parameters of M1 – M4 on the response (*Y1-Y4*) is presented as three-dimensional contour plot (Figure S7). Models M1-M4 can be used for the description of the influence of studied process parameters for the adsorption of DCF, respectively.

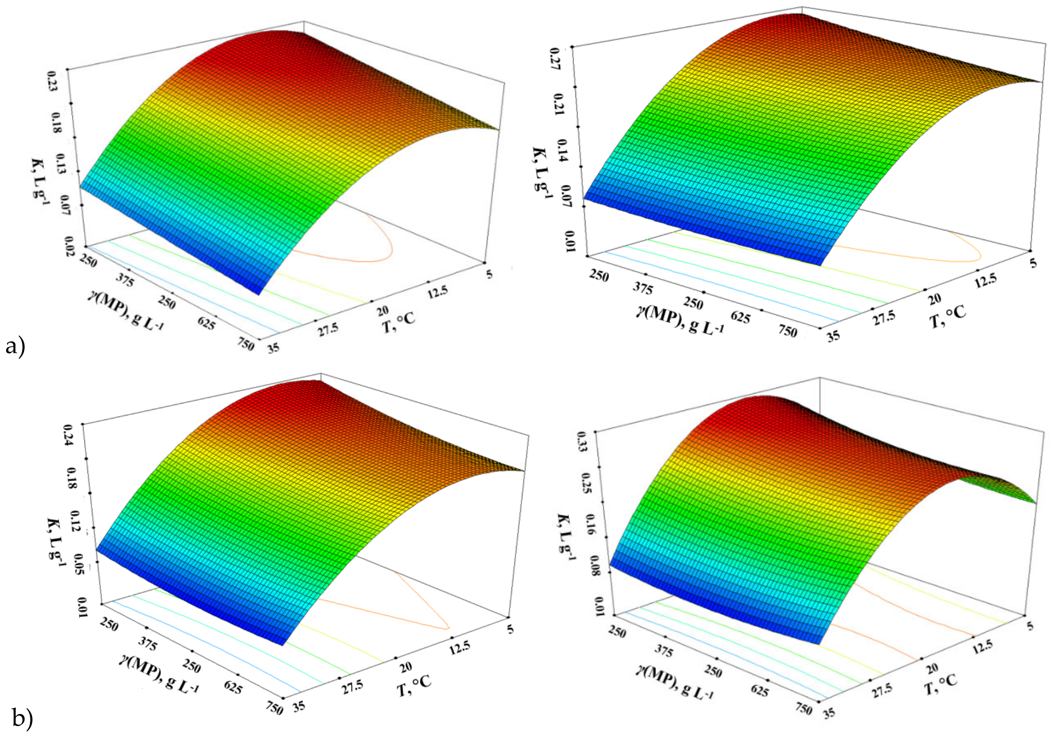

Figure S7. 3D response surface and contour diagrams showing the effects of the mutual interactions of initial T and γ (PET-MP) on the response (adsorption of DCF), for the samples: (**a**) PET B 0 (left), PET B 42 (right); (**b**) PET F 0 (left), PET F 42 (right).

Table S5. Physicochemical properties and molecular structure of PET and DCF.[5,6.]

Abbreviation	Molecular Structure	Molecular Formula	Cas No.	Molecular Weight (g mol^{-1})	Solubility in Water at 25°C (mg L^{-1})	Log K_{ow}	pK_a
PET	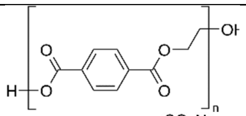	$C_{10}H_{12}O_6$	25038-59-9	50000–110000	-	-	-
DCF	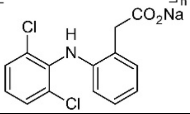	$C_{14}H_{10}Cl_2NNaO_2$	15307-79-6	318.1	2.37	4.51	4.15

References

[1] Almond, J.; Sugumaar, P.; Wenzel, M. N.; Hill, G.; Wallis, C. Determination of the carbonyl index of polyethylene and polypropylene using specified area under band methodology with ATR-FTIR spectroscopy. *e-Polymers* **2020**, *20,* 369–381.

[2] Brandon, J.; Goldstein, M.; Ohman, M. D. Long-term aging and degradation of microplastic particles: Comparing in situ oceanic and experimental weathering patterns. *Mar. Pollut. Bull.* **2016**, *110,* 299–308.

[3] Blum, D.J.W.; Suffet, I. H.; Duguet, J. P. Quantitative structure-activity relationship using molecular connectivity for the activated carbon adsorption of organic chemicals in water. *Water Res* **1994**, *28*, 687–699.

[4] Tomic, A.; Cvetnic, M.; Kovacic, M.; Kusic, H.; Karamanis, P.; Loncaric Bozic, A. Structural features promoting adsorption of contaminants of emerging concern onto TiO2 P25: experimental and computational approaches. *Environ. Sci. Pollut. Res.* **2022**, *29*, 87628–87644.

[5] pK_a (the acidic constant of DCF) and log K_{ow} (the octanol-water partition coefficient) data from *Pubchem* database: URL: https://pubchem.ncbi.nlm.nih.gov/compound/3033#section=Vapor-Pressure (11.3.2023.)

[6] Semalty, A.; Semalty, M.; Singh, D.; Rawat, M.S. Development and physicochemical evaluation of pharmacosomes of diclofenac. *Acta Pharm.* **2009**, *59*, 335-344.

Disclaimer/Publisher's Note: The statements, opinions and data contained in all publications are solely those of the individual author(s) and contributor(s) and not of MDPI and/or the editor(s). MDPI and/or the editor(s) disclaim responsibility for any injury to people or property resulting from any ideas, methods, instructions or products referred to in the content.
